# Artificial micro-cinderella based on self-propelled micromagnets for the active separation of paramagnetic particles†
†Electronic supplementary information (ESI) available: Experimental section; Fig. S1. Video S1–S5. See DOI: 10.1039/c3cc41962b



**DOI:** 10.1039/c3cc41962b

**Published:** 2013-04-29

**Authors:** Guanjia Zhao, Hong Wang, Samuel Sanchez, Oliver G. Schmidt, Martin Pumera

**Affiliations:** a Division of Chemistry and Biological Chemistry , School of Physical and Mathematical Sciences , Nanyang Technological University , 637371 , Singapore . Email: pumera@ntu.edu.sg ; Fax: +65-6791-1961; b Institute for Integrative Nanosciences , IFW Dresden , Helmholtzstrasse 20 , D-01069 Dresden , Germany; c Material Systems for Nanoelectronics , Chemnitz Technical University , Reichenhainer Strasse 70 , 09107 Chemnitz , Germany

## Abstract

In this work, we will show that ferromagnetic microjets can pick-up paramagnetic beads while not showing any interaction with diamagnetic silica microparticles for the active separation of microparticles in solution.

## Abstract 2

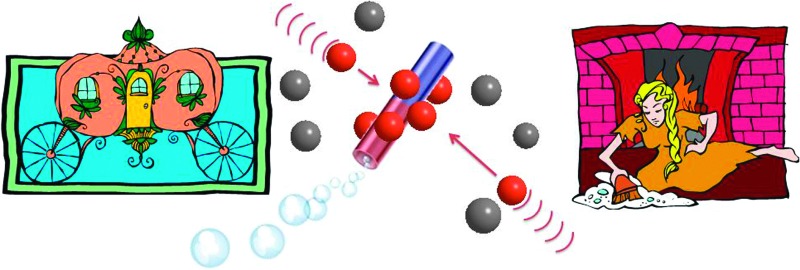
Ferromagnetic microjets can pick-up paramagnetic beads while not showing any interaction with diamagnetic silica microparticles for active separation of microparticles.

Synthetic nano- and micromotors have been gathering accumulated interest amongst the researchers in recent years.^1–6^ The successful fabrication of micromotors of various designs has been achieved and reported with a high reproducibility. Different kinds of motors have also emerged in the literature, such as bimetallic microwires,^7^ Janus spheres,^8^ screw-shaped motors,^9^ and nano/microtubes.^10^ The propulsion of such motors can be attributed to different types of mechanisms, including self-electrophoresis,^11^ diffusiophoresis^12^ or bubble propulsion.^2^ Recent efforts have been mainly focused on bubble-propelled microjets and their applications in different fields. These microdevices are self-propelled by the decomposition of fuel, typically hydrogen peroxide, on its inner surface such as catalytic Pt metal.^2^

One of the main applications of micromotors is the directed and selective pick-up of cargo particle.^13^ This has been achieved *via* the (i) mechanical attachment to the microjet tip,^14^ (ii) electrostatic interaction between the negatively charged polypyrrole segment of nanorod microjets and the positively charged cargo particle,^15^ (iii) usage of a biorecognition element, such as single stranded DNA, protein molecules,^16,17^ or (iv) chemical bonds.^18^ Here we wish to present for the first time the highly selective pick-up of cargo by microjet engines with no surface modifications but with magnetic functionality. Such microjet engines are based on the hydrogen peroxide propelled microtubes containing a permanent magnetic moment that are able to selectively pick up paramagnetic beads from their mixture with diamagnetic microparticles (Scheme 1) in the absence of an external magnetic field.

**Scheme 1:**
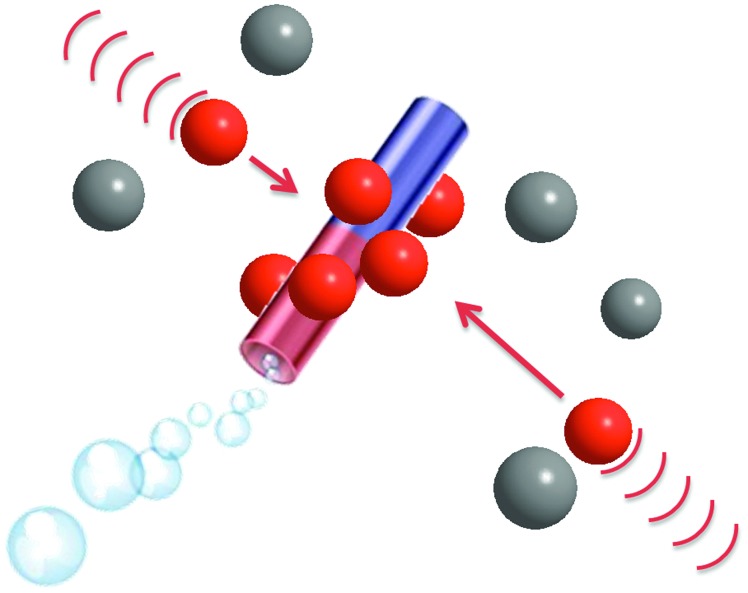
Self-propelled microjets with a permanent magnetic moment attract paramagnetic particles (brown, dynabeads) and carry them while not influencing the diamagnetic (grey, SiO_2_) particles.

Rolled-up microjets of diameter 5 μm and length 50 μm are fabricated by the thin film deposition method as described previously.^19^ The rolled-up microjets consist of a layered structure of Ti, Fe, Cr and Pt nanomembranes with thicknesses of 3, 5, 5 and 1 nm, respectively (for details on fabrication, see ESI†; for a SEM image of microjets, see Fig. S1, ESI†). It should be noted that upon exposure of such microtubes to an external magnetic field with an intensity of ∼270 mT for several seconds, the microtube becomes ferromagnetic with its own magnetic moment.^20^ Such a “micromagnet” tube responds to weak changes in the external magnetic field by changing its orientation.^20^

When such a microtube is immersed into a solution containing hydrogen peroxide, the hydrogen peroxide decomposes causing the microjet to propel forward.

The magnetized microjets swimming in H_2_O_2_ solution behave in a similar way to self-propelled micromagnets, attracting paramagnetic micro-objects. In Fig. 1A (and related Video S1, see ESI†) the movement of a non-magnetized (that is, not containing permanent magnetic moment) microjet in a mixture containing 2.7 μm diameter paramagnetic beads (Dynal®) can be seen. Those non-magnetized microjets do not show any interaction with the paramagnetic beads in such case, moving at an average velocity of 130 μm s^–1^. When the Fe-segment containing microjet is exposed to a magnetic field of neodymium for 10 seconds, it is able to attract and carry a large volume (>30 beads) of paramagnetic beads attached to its surface (Fig. 1B and corresponding Video S2, ESI†). Differently, we observed that the non-magnetized microjets are not capable of any interaction with the paramagnetic beads while the magnetized microjets are capable of on-the-fly capture of the paramagnetic beads (Fig. 1C, and corresponding Video S3, ESI†). We used Dynal® paramagnetic beads with a streptavidin functionalized surface.

**Fig. 1:**
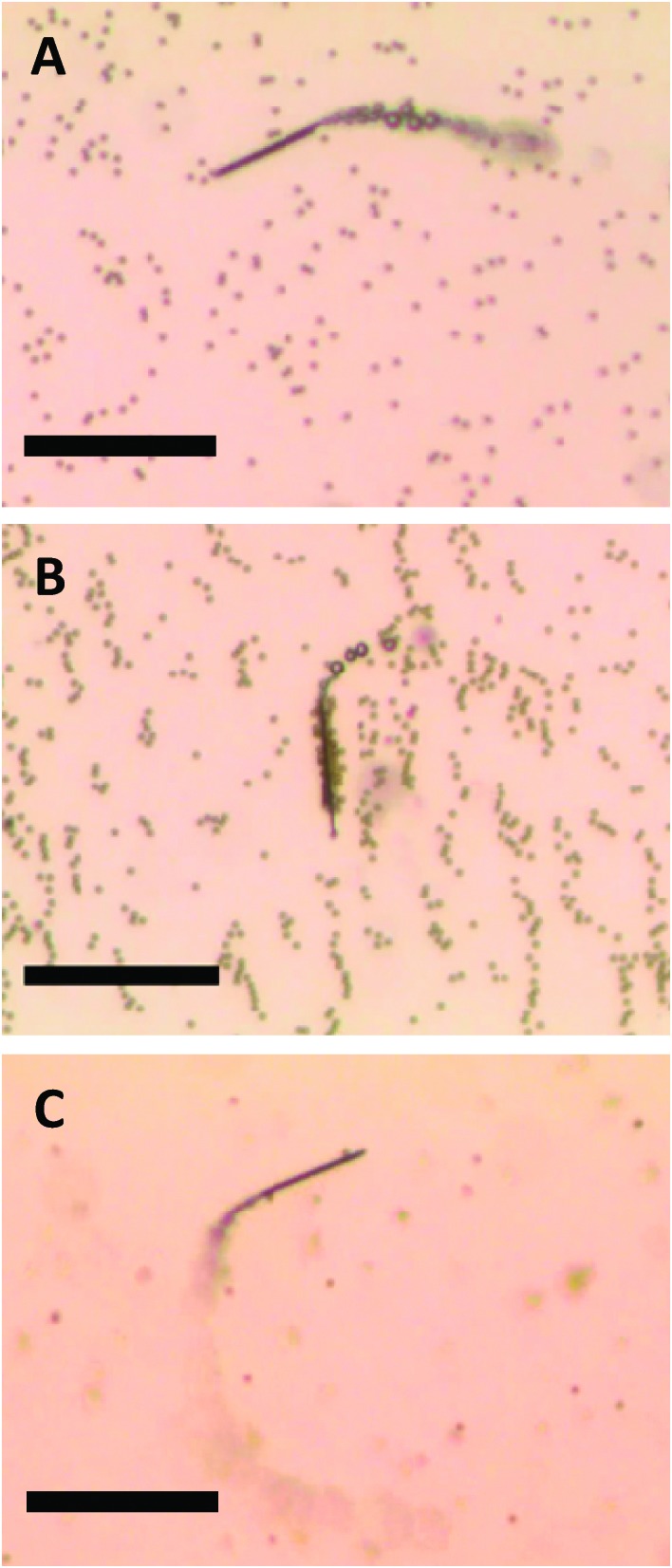
Movement of (A) non-magnetized and (B) magnetized microjet engines in a paramagnetic beads suspension. (C) On-the-fly pick-up of paramagnetic beads by magnetized microjets. Scale bar of 50 μm. Conditions: 6% H_2_O_2_, 1% SDS, beads concentration approximates to 3 × 10^5^ μL^–1^.

The surface of such beads can be functionalized with practically any biomolecule which gives it a great potential in highly tailored bioapplications. Note that such an on-the-fly paramagnetic bead capture is possible in the absence of an external magnetic field as the micromotor acts as a permanent magnet.

The ordered “chain” structures of paramagnetic beads seen in Fig. 1B are remnant structures after application of an external magnetic field in order to magnetize microjet engines. All videos S1–S3 (ESI†) and Fig. 1 were taken in the absence of an external magnetic field.

We noted that self-electrophoretically powered nanorods were also found to be capable of picking up beads loaded with Fe_3_O_4_. However, such pick-up and the hold of microbeads were demonstrated to occur only under a constant influence of an external magnetic field,^21^ which is fundamentally different from the example presented here in our work, where the microjet engine is magnetized and is able to carry paramagnetic beads in the absence of an external magnetic field.

The ability to selectively pick-up paramagnetic particles from their mixtures with diamagnetic particles was explored with the magnetic functionality of self-propelled microjets. Fig. 2 (and corresponding Video S4, ESI†) shows the movement of self-propelled magnetized microjets (without further influence of the external magnetic field) in a mixture of paramagnetic dynabeads and diamagnetic silica microparticles. The magnetized microjets were able to selectively pick-up the paramagnetic beads while not attracting the diamagnetic SiO_2_ microparticles at the same time. Control experiments using non-magnetized microjet engines showed that such non-magnetized microjets do not distinguish between the paramagnetic and diamagnetic particles and move through their dispersion without any sign of magnetic interactions (Fig. 3 and corresponding Video S5, ESI†).

**Fig. 2:**
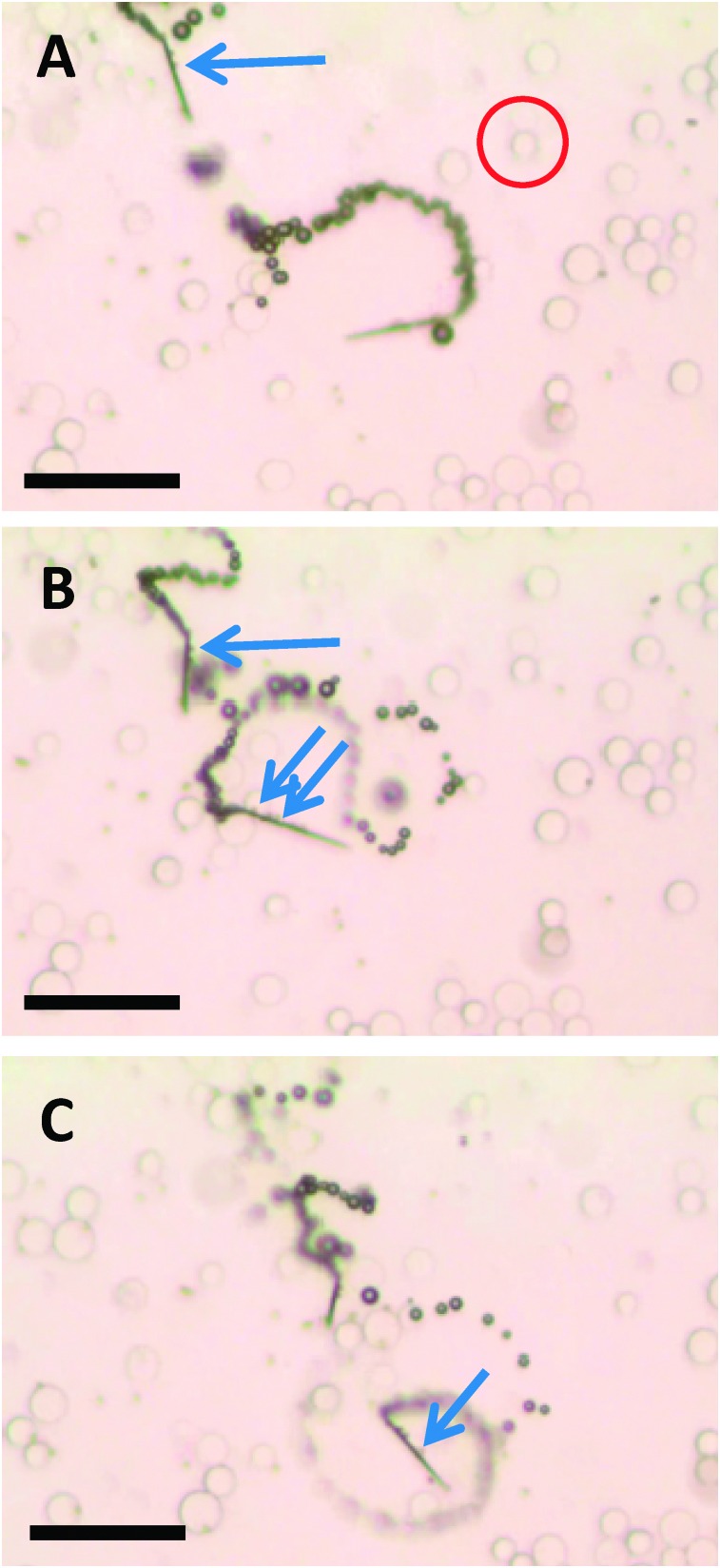
Magnetized microjets are capable of selective pick-up of paramagnetic beads (see arrow, dark spots: dynabeads of diameter of 2.7 μm) while diamagnetic beads (see example inside the red circle, SiO_2_ microparticles with a maximum diameter of 20 μm). A, B and C represent time 0, 2 and 4 s of time frame. Scale bar of 50 μm. Conditions: 6% H_2_O_2_, 1% SDS, beads concentration approximates to 3 × 10^4^ μL^–1^ for the silica beads and 1 × 10^4^ μL^–1^ dynabeads.

**Fig. 3:**
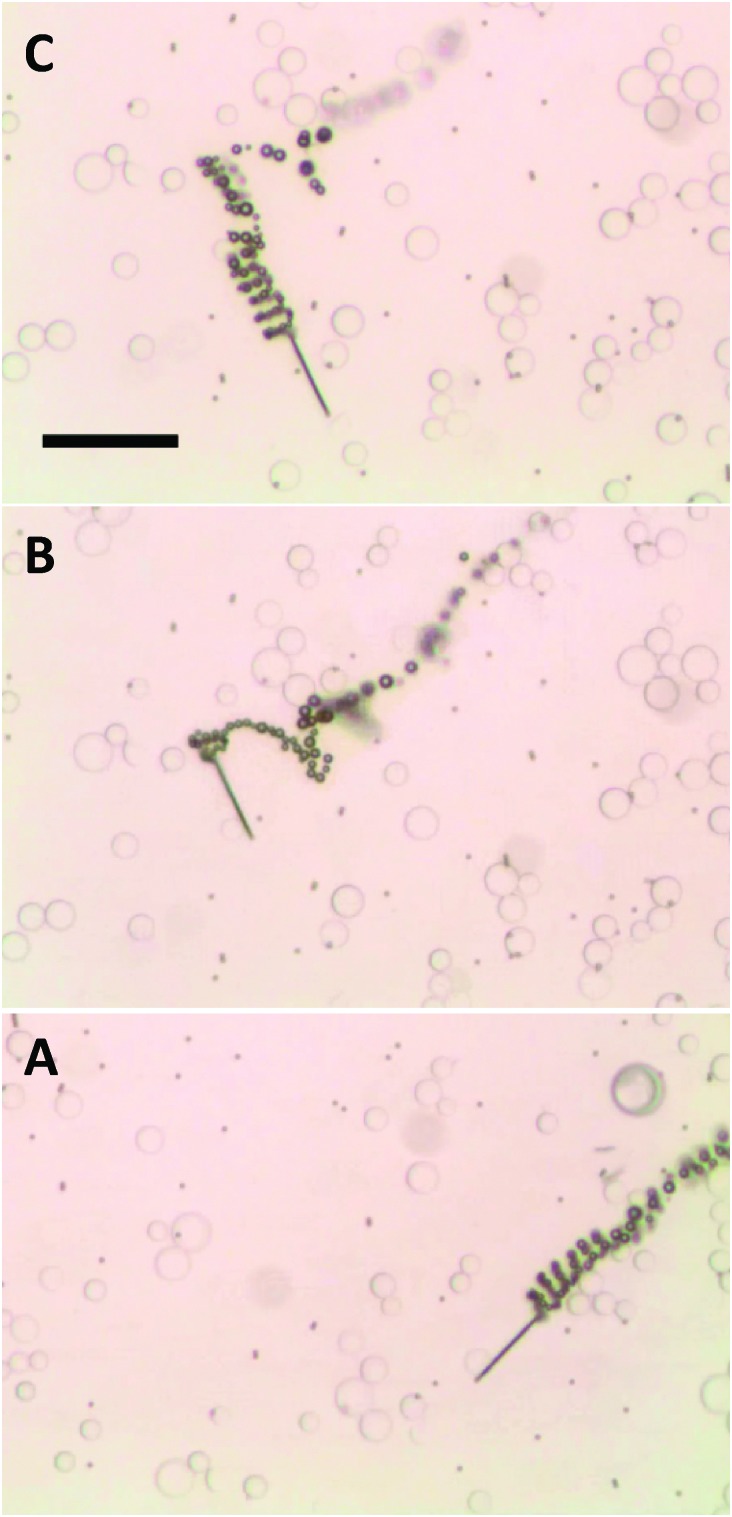
Non-magnetized microjets are *not* capable of pick-up of paramagnetic beads (dark spots) from their mixture with silica beads (transparent beads), moving through their mixture and ignoring them all. A, B and C represent time 0, 3 and 5 s of time frame. Scale bar of 50 μm. Conditions: 6% H_2_O_2_, 1% SDS, beads concentration approximates to 3 × 10^4^ μL^–1^ for the silica beads and 1 × 10^4^ μL^–1^ dynabeads.

In summary, we have demonstrated that the devices made of magnetized rolled-up microtubes containing Fe nanomembranes behave as small self-propelled micromagnets that are capable of actively picking-up and carrying paramagnetic cargo without the influence of any external magnetic field. Such self-propelled micromagnets are capable of distinguishing the paramagnetic microparticles from the diamagnetic ones, and thus selectively pick up the paramagnetic ones. These self-propelled microjets are expected to have applications in various micromotor-based assays.

M.P. thanks NTU-JSPS and NAP (NTU) funds for support. S.S. and O.G.S. thank the Volkswagen Foundation (project number 86362). S.S. thanks the European Research Council (ERC) for Starting Grant (LT-NRBS).

## References

[cit1] Paxton W. F., Sundararajan S., Mallouk T. E., Sen A. (2006). Angew. Chem., Int. Ed..

[cit2] Mei Y. F., Solovev A. A., Sanchez S., Schmidt O. G. (2011). Chem. Soc. Rev..

[cit3] Sanchez S., Pumera M. (2009). Chem.–Asian J..

[cit4] Fisher P., Ghosh A. (2011). Nanoscale.

[cit5] Ebbens S. J., Howse J. R. (2010). Soft Matter.

[cit6] Wang J. (2009). ACS Nano.

[cit7] Kline T. R., Paxton W. F., Mallouk T. E., Sen A. (2005). Angew. Chem., Int. Ed..

[cit8] Baraban L., Makarov D., Streubel R., Monch I., Grimm D., Sanchez S., Schmidt O. G. (2012). ACS Nano.

[cit9] Peyer K. E., Zhang L., Nelson B. J. (2013). Nanoscale.

[cit10] Mei Y. F., Huang G., Solovev A. A., Urena E. B., Monch I., Ding F., Reindl T., Fu R. K. Y., Chu P. K., Schmidt O. G. (2008). Adv. Mater..

[cit11] Pumera M. (2010). Nanoscale.

[cit12] Ibele M., Mallouk T. E., Sen A. (2009). Angew. Chem., Int. Ed..

[cit13] Patra D., Sengupta S., Duan W., Zhang H., Pavlick R., Sen A. (2013). Nanoscale.

[cit14] Sanchez S., Solovev A. A., Harazim S. M., Schmidt O. G. (2011). J. Am. Chem. Soc..

[cit15] Sundararajan S., Lammert P. E., Zudans A. W., Crespi V. H., Sen A. (2008). Nano Lett..

[cit16] Kagan D., Campuzano S., Balasubramanian S., Kuralay F., Flechsig G., Wang J. (2011). Nano Lett..

[cit17] García M., Orozco J., Guix M., Gao W., Sattayasamitsathit S., Escarpa A., Merkoçi A., Wang J. (2013). Nanoscale.

[cit18] Sundararajan S., Sengupta S., Ibele M. E., Sen A. (2010). Small.

[cit19] Solovev A. A., Sanchez S., Pumera M., Mei Y. F., Schmidt O. G. (2010). Adv. Funct. Mater..

[cit20] Zhao G., Sanchez S., Schmidt O. G., Pumera M. (2012). Chem. Commun..

[cit21] Kagan D., Laocharoensuk R., Zimmerman M., Clawson C., Balasubramanian S., Kang D., Bishop D., Sattayasamitsathit S., Zhang L., Wang J. (2010). Small.

