# Supplementary material for: Artificial micro-cinderella based on self-propelled micromagnets for the active separation of paramagnetic particles
Source: Chem Commun (Camb). 2013 Apr 29;49(45):5147–9. doi: 10.1039/c3cc41962b (PMC3782880; doi:10.1039/c3cc41962b)
Supplement: Supplementary file 1 [file CC-049-c3cc41962b-s001.pdf]

Electronic Supplementary Material (ESI) for Chemical Communications
This journal is © The Royal Society of Chemistry 2013

Electronic Supplementary Information

Artificial Micro-Cinderella Sorting out Peas and Lentils: Self-Propelled Micromagnets for Picking up the Paramagnetic Beads from Their Mixtures with Diamagnetic Particles

Guanjia Zhao[a], *Hong Wang*[a], *Samuel Sanchez*[b], *Oliver G. Schmidt*[b], *Martin Pumera*[a]*

[a] G. Zhao, Hong Wang, Prof. M. Pumera*,
Division of Chemistry & Biological Chemistry
School of Physical and Mathematical Sciences
Nanyang Technological University
Singapore 637371
Fax: (65) 6791-1961
E-mail: pumera@ntu.edu.sg

[b] Dr. Samuel Sanchez, Prof. Oliver G. Schmidt,
Institute for Integrative Nanosciences, IFW Dresden,
Helmholtzstrasse 20, D-01069 Dresden, Germany.

This journal is © The Royal Society of Chemistry 2013

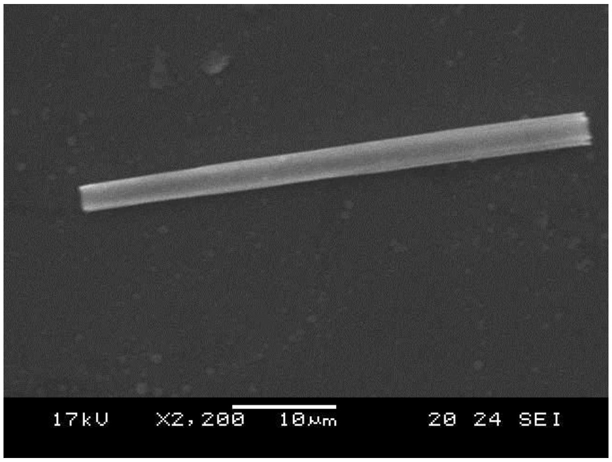

Figure S1. Scanning electron micrograph of microjet.

This journal is © The Royal Society of Chemistry 2013

Experimental Section

Materials and Apparatus. Hydrogen peroxide (27% wt., Lot # 10151507) was purchased from Alfa Aesar, Singapore. Sodium dodecyl sulfate (SDS, Lot # 079K0335) was purchased from Sigma-Aldrich. The magnetic M-270 Dynabeads (diameter of 2.8 μm) were purchased from Invitrogen. Silica beads (diameter of 20 μm) were purchased from Alfa Aesar (Lot # C19W016). Chemicals were used as received and solutions were prepared using ultrapure water (18.2 MΩ cm) from a Millipore Milli-Q purification system. Optical microscope videos and images were obtained with Nikon Eclipse 50*i* microscope. Video sequences were processed with Nikon NIS-Elements^{TM} software.

Preparation of Rolled-up Microtubes: Fabrication of Ti/Fe/Cr/Pt microtubes was carried out with the previously reported protocol.[1] Briefly, a layer of AR-P 3510 photoresist was spin-coated on silicon wafers (1.5 inch) at 3500 rpm for 35 s. After that, a soft bake using a hotplate was carried out at 90°C for 1 min. The wafer was then exposed to UV light with a Karl Suss MA-56 mask aligner (410–605 nm). The exposed patterns of the photoresist were developed in an AR300-35/H_2O solution (1:1). Microtubes were obtained by a tilted deposition at an angle of about 60° (measured from the horizontal axis) on the photoresist. Metal films with thicknesses of 3 nm (Ti), 5 nm (Fe) and 5 nm (Cr) were deposited layer by layer on the tilted samples. Using magnetron sputtering, a layer of Pt with 1 nm thickness was deposited onto the Ti/Fe/Cr samples. Rolling-up of films into tubular structure was done by immersing the sample into acetone, as it could selectively etch the photoresist layer. Finally, supercritical point drying was used to keep the tubes from collapsing due to high fluid surface tension.

This journal is © The Royal Society of Chemistry 2013

Micromotor operations. Motion studies of the micromotors in beads surroundings were carried out in an aqueous solution containing 6 wt% of hydrogen peroxide at constant surfactant concentrations (1 wt% of SDS). Solutions of the M-270 Dynabeads and silica particles were diluted with ultrapure water. A mixture of the micromotors (5 µL), SDS (1 wt %), H_2O_2 (6 wt %) and the pre-diluted bead solution (1 µL) was applied on a glass slide freshly cleaned with nitrogen gas. Magnetic field was applied by placing permanent neodymium magnet about 1 cm from the glass slide in the horizontal plane.

---

1 A. A. Solovev, S. Sanchez, M. Pumera, Y. Mei, O. G. Schmidt, *Adv. Func. Mater.* 2010, **20,** 2430.
